# Cellulase Production by Ultraviolet-Derived Mutant *Trichoderma* sp. Mut-4 Under Submerged Fermentation: Parameter Optimization and Large-Scale Application

**DOI:** 10.3390/ijms26168000

**Published:** 2025-08-19

**Authors:** Iksu Ha, Seungjun Kim, Yun-Yeong Lee, Junseo Lee, Jeonghee Yun

**Affiliations:** 1 Department of Forest Products and Biotechnology, Kookmin University, Seoul 02707, Republic of Korea; gk257252@kookmin.ac.kr (I.H.); yunlee529@kookmin.ac.kr (Y.-Y.L.); lmn9022@kookmin.ac.kr (J.L.); 2 Forest Carbon Graduate School, Kookmin University, 77 Jeongneungro, Seongbukgu, Seoul 02707, Republic of Korea

**Keywords:** biorefinery, UV-derived mutant, cellulase production, bioprocessing, high activity

## Abstract

This study aimed to optimize the parameters, including medium formulations and culture conditions, for submerged fermentation (SmF) systems using a mutant strain of *Trichoderma* sp., Mut-4. Optimization was performed using the one-factor-at-a-time (OFAT) method to enhance cellulase activity and productivity. Parameters such as the blending ratio of carbon sources, type of nitrogen source, and initial pH were evaluated for their effects on enzyme activity and productivity. The optimal conditions were determined to be as follows: a 3:1 Avicel-to-cellulose ratio, yeast extract as the nitrogen source, and an initial pH of 5.5. Under these conditions, cellulase production was initiated earlier, and the activity of all cellulase components, along with protein concentration, increased by 1.17- to 1.43-fold at the flask scale and by 1.3- to 2.0-fold at the reactor scale. These results demonstrate the superior activity and productivity of Mut-4 under optimized conditions, highlighting its potential for application in large-scale cellulase production.

## 1. Introduction

Lignocellulosic biomass (LCB) has attracted significant interest as a sustainable alternative feedstock in response to growing concerns about climate change and the depletion of fossil resources [1]. LCB is one of the most abundant renewable resources globally, encompassing materials such as agricultural residues, forestry waste, and other plant-derived biomasses [2,3]. The use of LCB in biorefineries to produce fuels and chemicals such as bioethanol and bioplastics offers a promising route to reduce the current reliance on petrochemicals [4,5,6,7]. Cellulose, which is the primary component of LCB, is the most abundant biopolymer on Earth and consists of linear chains of β-1,4-linked d-glucose [8]. Therefore, effective cellulose degradation into fermentable sugars is a critical step for converting biomass into biofuels and value-added products [9,10,11]. However, because native cellulose that is present in the plant matter is tightly associated with hemicellulose and lignin in a recalcitrant matrix, the use of pretreatment and saccharification steps is necessary to release fermentable sugars from them [12,13].

Cellulose can be hydrolyzed using either harsh chemicals or specific enzymes [14]. Enzymatic saccharification using cellulase has emerged as the preferred method for depolymerizing cellulose because acid hydrolysis requires extreme conditions and generates inhibitory byproducts, such as furfural and 5-Hydroxymethylfurfural (HMF), whereas enzymatic hydrolysis operates under milder conditions and generates fewer toxins [15,16]. Cellulase consists of three major synergistic enzymes: endoglucanase (EG) randomly cleaves the internal β-1,4-glycosidic bonds within cellulase chains, cellobiohydrolase (CBH) releases cellobiose units from the ends of the cellulose chains, and β-glucosidase (BGL) converts cellobiose into glucose [17]. These enzymes act cooperatively to efficiently convert cellulose into soluble sugars. Filamentous fungi, primarily species of *Trichoderma*, *Aspergillus*, and *Penicillium*, are the primary industrial producers of cellulases [18,19]. Although cellulases are used in a wide range of applications, including biofuels, textiles, and food processing, their production cost remains a major bottleneck [20,21]. Therefore, intensive research has focused on developing strategies to improve cellulase productivity and obtain high activity enzyme systems [20,22,23].

Submerged fermentation (SmF) is widely used in industrial cellulase production because of its controllability and scalability [24]. However, SmF often yields lower enzyme production than solid-state fermentation (SSF), particularly when insoluble lignocellulosic substrates are used [25]. To improve cellulase productivity under SmF, researchers have optimized key parameters such as the carbon and nitrogen sources and initial pH [22,26,27]. Moreover, recent studies have introduced solid support materials into SmF to provide attachment sites for fungi, thereby mimicking the SSF microenvironment and enhancing enzyme secretion [28]. Among the various support materials that are available for this purpose, biochar has been widely used as a promising additive to improve fungal growth and enzyme production in SmF systems [29]. In our previous study, we demonstrated that biochar supplementation in *Trichoderma* cultures significantly improved cellulase productivity [23]. The activities of EG, BGL, and CBH increased by 1.4- to 2.1-fold at the flask scale and by up to 12-fold in bioreactor systems [23]. These improvements were attributed to the ability of biochar to foster a favorable growth environment, as its porous structure can harbor fungal cells and facilitate better access to insoluble substrates.

In addition to process-based strategies (such as the use of support materials) the efforts aimed at enhancing cellulase activity have also focused on strain improvement through genetic engineering or classical mutagenesis. For example, via successive rounds of random mutagenesis and selection, industrial *Trichoderma reesei* strains that secrete over 100 g·L^−1^ of cellulolytic enzymes have been developed, thereby far surpassing the output of the wild-type organism [30]. These enhanced strains form the basis of many commercial cellulase preparations. Based on this approach, our research group previously used ultraviolet (UV)-induced mutagenesis to obtain a mutant cellulase-producing strain, *Trichoderma* sp. Mut-4. The sequence of this strain has been deposited in the NCBI GenBank database under the accession number PV565723 [31]. *Trichoderma longibrachiatum* KMF006 (KCTC13500BP), which is a patented strain officially deposited under the accession number KCTC13500BP in the Korean Collection for Type Cultures (KCTC), was subjected to UV irradiation (302 nm) for various periods and distances. Among the resulting strains, Mut-4, which was irradiated for 4 min at a distance of 4 cm, exhibited cellulase activities 1.26- to 1.55-fold that of the parental strain [31]. Based on these previous results, the present study employed the UV-derived Mut-4 mutant strain of *Trichoderma* sp. which had previously yielded enhanced cellulase productivity. Using this strain, we aimed to optimize the culture conditions, specifically the carbon and nitrogen sources and the initial pH, within our newly developed biochar-supplemented SmF system [23]. The optimization was based on a one-factor-at-a-time (OFAT) approach to identify the effects of individual conditions that maximized enzyme activity and productivity. The optimized process was then applied at the bioreactor scale to assess its feasibility for larger-scale enzyme production. Thus, this study built upon previous findings by integrating a high-performing mutant into a solid support strategy and further validating their combined effects under scaled-up fermentation conditions.

## 2. Results and Discussion

### 2.1. Effect of the Carbon Sources on Cellulase Activity

**Figure 1:**
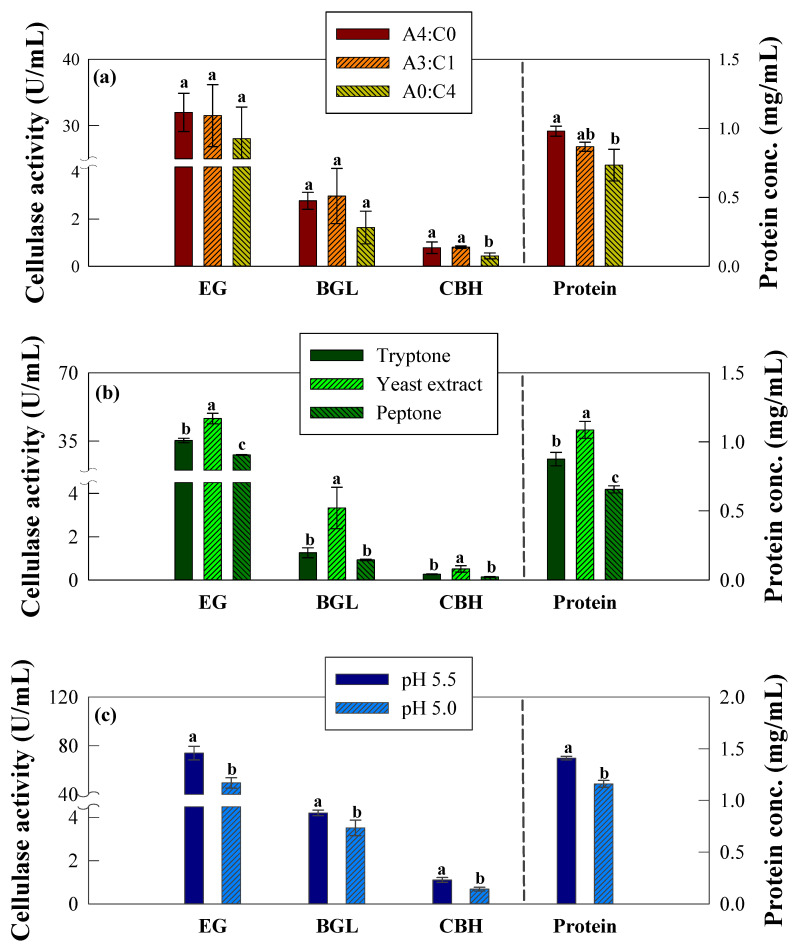
Maximum enzymatic activities (EG, BGL, and CBH) and protein concentration (mg mL^−1^) measurements of Mut-4 in response to different optimization variables. (**a**) Carbon source ratios: A4:C0, Avicel:Cellulose = 4:0; A3:C1 = Avicel:Cellulose = 3:1; and A0:C4 = Avicel:Cellulose = 0:4. (**b**) Nitrogen source: tryptone, yeast extract, and peptone. (**c**) Initial pH: pH 5.5, pH 5.0, and pH 4.5. The measured enzymatic activities were those of EG (endoglucanase), BGL (β-glucanase), and CBH (cellobiohydrolase). Error bars represent standard deviation (SD) from six measurements (*n* = 6). Statistical significance was determined by one-way ANOVA followed by Tukey’s HSD test (*p* < 0.05). Different letters indicate significant differences among the groups.

The time profiles of cellulase activities and protein concentrations under different blending ratios of the carbon sources (Avicel and cellulose), which illustrated the temporal dynamics of the system, are reported in Figure S1. All activities and protein concentrations exhibited similar trends, with a rapid increase occurring over days 10–14 and reaching maximum values on days 14–18. The maximum enzymatic activities and protein concentrations are presented in Figure 1a and Table S1. Across all tested blending ratios of Avicel and cellulose, no statistically significant differences (*p* > 0.05) were observed in the EG and BGL activities, which ranged from 28.0–32.0 and 1.6–3.0 U·mL^−1^, respectively. Similarly, protein concentrations remained comparable (0.73–0.98 mg·mL^−1^). Only the CBH activity under the A0:C4 condition showed a significant decrease (0.44 U·mL^−1^) compared to other ratios (0.79–0.81 U·mL^−1^).

**Figure 2:**
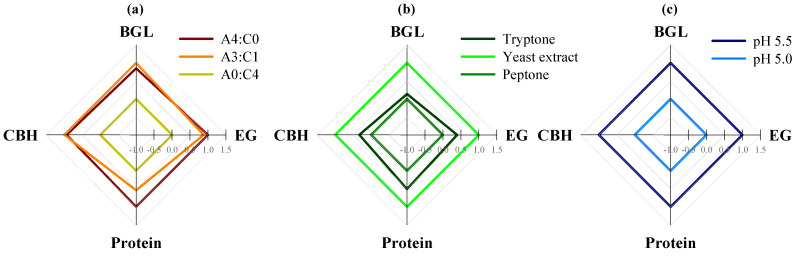
Maximum reaction rates of enzymatic activities (EG, BGL, and CBH) of Mut-4 in response to different optimization parameters. (**a**) Carbon source: A4:C0, Avicel:Cellulose = 4:0; A3:C1 = Avicel:Cellulose = 3:1; and A0:C4 = Avicel:Cellulose = 0:4. (**b**) Nitrogen source: tryptone, yeast extract, and peptone. (**c**) Initial pH: pH 5.5, pH 5.03, and pH 4.5. The measured enzymatic activities were those of EG (endoglucanase), BGL (β-glucanase) and CBH (cellobiohydrolase). Visualization was performed with values scaled using the min–max normalization.

**Figure 3:**
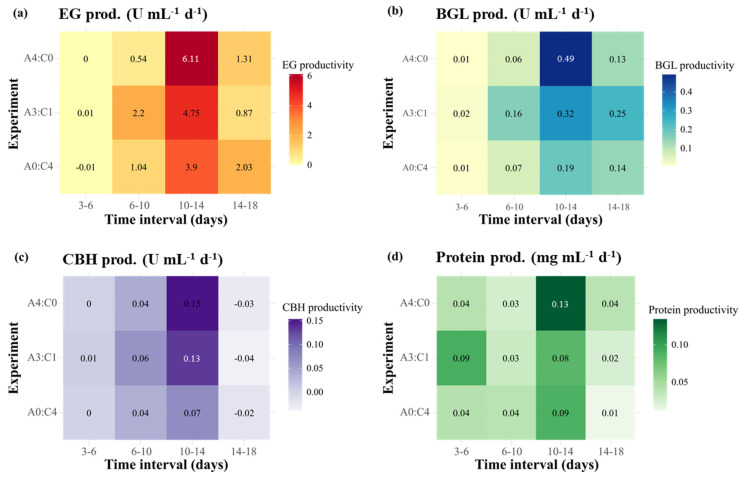
Time-interval productivity of enzymatic activities (EG, BGL, and CBH) and protein concentration of Mut-4 in response to different blending ratios of carbon sources. (**a**) EG (endoglucanase), (**b**) BGL (β-glucanase), (**c**) CBH (cellobiohydrolase), and (**d**) protein concentration. The blending ratios are presented as follows: A4:C0, Avicel:Cellulose = 4:0; A3:C1 = Avicel:Cellulose = 3:1; and A0:C4 = Avicel:Cellulose = 0:4.

The maximum production rates of enzymatic activities and protein concentrations for the different blending ratios are shown in Figure 2a and Table S2. The values reported in Figure 2a were scaled using min–max normalization. With the exception of protein concentration, the maximum production rates under the A4:C0 and A3:C1 conditions were almost identical, with EG, BGL, and CBH activities ranging from 1.75–1.78, 0.15–0.17, and 0.056–0.058 U·mL^−1^·d^−1^, respectively. In contrast, lower production rates across all parameters were observed under the A0:C4 condition. The productivity in terms of enzymatic activities and protein concentrations over defined time intervals is presented in Figure 3. In this context, productivity represented the rate of increase in enzyme activity or protein concentration per day, as calculated from the difference between two consecutive time points. In all experimental groups, productivity was highest on days 10–14, particularly for EG (Figure 3a), which exhibited a steep increase compared with earlier stages. Among the tested groups, the highest overall productivity was observed under the A4:C0 condition, with peak values occurring during days 10–14. Interestingly, relatively higher productivity was observed under the A3:C1 condition at earlier intervals, with a noticeable surge beginning during days 6–10, suggesting a more rapid onset of enzyme production compared to the other groups. In contrast, the lowest overall productivity was observed under the A0:C4 condition, although it surpassed the A4:C0 condition in EG productivity during days 14–18. For the other enzymes and protein concentration, however, the A0:C4 condition consistently showed lower productivity throughout the entire experimental period.

These results suggest that the blending ratio of Avicel and cellulose significantly affects the cellulase production patterns and protein concentration. The highest overall productivity was observed under the A4:C0 condition, while higher enzymatic productivity at earlier stages was observed under the A3:C1 condition. In contrast, an increase in the proportion of cellulose tended to reduce the overall enzymatic activities and protein concentrations. Moreover, no statistically significant differences were observed between A4:C0 and A3:C1 in terms of the timing and maximum values of enzymatic activities, suggesting that a partial replacement of Avicel with cellulose does not compromise the cellulase production efficiency.

Avicel is widely recognized as a potent inducer of cellulase expression because of its highly crystalline structure, which activates the fungal enzymatic system via substrate-specific induction pathways [32,33]. However, the high cost of Avicel restricts its application as the sole carbon source for large-scale processes. In contrast, cellulose is more cost-effective but exhibits a relatively weaker induction capacity. In this study, the crystallinity of Avicel and cellulose, which were used as carbon sources, was 75.52% and 54.00%, respectively (Table S3), reflecting the higher crystalline structure of Avicel compared with cellulose. Our results demonstrated that the combination of Avicel with cellulose at defined ratios, particularly in the A3:C1 condition (Avicel:cellulose = 3:1), achieved an optimal balance between efficient enzyme induction and cost efficiency. The Avicel-supplemented conditions (A4:C0 and A3:C1) yielded markedly higher cellulolytic activities and total protein concentrations compared with the A0:C4 condition, which included cellulose alone as the carbon source. The maximum activities of EG, BGL, and CBH in the A0:C4 condition were markedly lower, indicating insufficient induction of the full cellulolytic system. This trend aligned with previous reports suggesting that the absence of crystalline cellulose reduces the induction of key cellulases, such as CBH [32,34].

In the A0:C4 condition, the activities of BGL and CBH were significantly lower than those observed in the Avicel-supplemented cultures. In turn, EG exhibited a comparable level of activity to that of the remaining experimental groups. This was presumably attributable to the constitutive low-level expression of EG and its partial accessibility to the amorphous regions of cellulose, which allowed limited initial hydrolysis. In contrast, the induction of BGL and CBH requires stronger and more sustained signals, which is typically provided by crystalline substrates such as Avicel [35]. Thus, the highly crystalline structure of Avicel enhanced CBH activity, which promoted the accumulation of cello-oligosaccharides such as cellobiose, which acts as an inducer for downstream enzymes, including BGL [36,37]. However, the lower crystallinity of cellulose compared with Avicel may lead to reduced CBH secretion, thereby limiting the accumulation of oligosaccharide inducers such as cellobiose and consequently hindering the induction of BGL.

Notably, the A3:C1 condition outperformed even the A4:C0 condition in terms of productivity timing. The addition of the amorphous structure in A3:C1 appeared to release soluble oligosaccharides early, thus accelerating the induction phase [38]. Once induction commenced, the remaining Avicel functioned as a long-lasting substrate for sustained enzyme biosynthesis [38]. This dual-phase benefit, i.e., early induction and extended productivity, highlights the synergistic interplay between the crystalline and amorphous cellulose components. The substrate’s microstructure and degree of crystallinity were also key factors that affected the enzymatic profiles. The A3:C1 condition exhibited enzymatic activity levels that were comparable to those of the Avicel-dominant A4:C0 condition. However, it notably induced earlier secretion of EG and BGL, reflecting the synergistic effect of the crystalline and amorphous cellulose components.

From a process engineering perspective, the A3:C1 condition reduces Avicel use by 25% while retaining comparable enzymatic output [36,39]. This partial substitution addresses the challenge of high induced costs in industrial settings and reflects a scalable and economically viable model for cellulase production. In conclusion, the A3:C1 condition emerged as the most effective carbon source blend, as it yielded a favorable balance between enzyme productivity and economic feasibility. Therefore, it was selected as the optimal carbon source condition for subsequent cellulase production processes.

### 2.2. Effect of Nitrogen Sources on Cellulase Activity

The time profiles obtained under different nitrogen sources exhibited marked effects on cellulase activity (Figure S2). All experimental groups exhibited an initial lag phase up to days 10–14. However, the yeast extract condition maintained relatively higher activities compared with the remaining groups during the lag phase. The maximum activities and protein concentrations are reported in Figure 1b and Table S1. The yeast extract condition exhibited significantly higher enzymatic activities compared with the tryptone and peptone conditions (*p* < 0.05). Among the tested nitrogen sources, yeast extract resulted in the highest enzymatic activities, with peak values of 46.54, 3.33, and 0.51 U·mL^−1^ for EG, BGL, and CBH, respectively. In contrast, lower activities were observed with tryptone and peptone. Yeast extract also supported the highest level of total secreted protein (1.09 mg·mL^−1^), significantly higher than those obtained with the other nitrogen sources (0.66 to 0.87 mg·mL^−1^).

The highest maximum production rates observed for the three enzymes were obtained in the yeast extract condition (Figure 2b, Table S2). The values depicted in Figure 2b were scaled using min–max normalization. The activities of most enzymes and protein concentrations generally followed the trend of yeast extract > tryptone > peptone. However, the BGL activity in the tryptone and peptone groups exhibited a distinct pattern, with nearly identical production rates observed between the two conditions (Figure 2b). In the Table S2, the maximum EG production rate with yeast extract was 1.32-fold higher than that with tryptone and 1.67-fold higher than that with peptone. Similarly, the peak production rates of BGL and CBH with yeast extract (0.185 and 0.029 U·mL^−1^·d^−1^) were 2–3-fold higher than those obtained with tryptone and peptone. The protein production rate was also higher with yeast extract than with tryptone or peptone.

**Figure 4:**
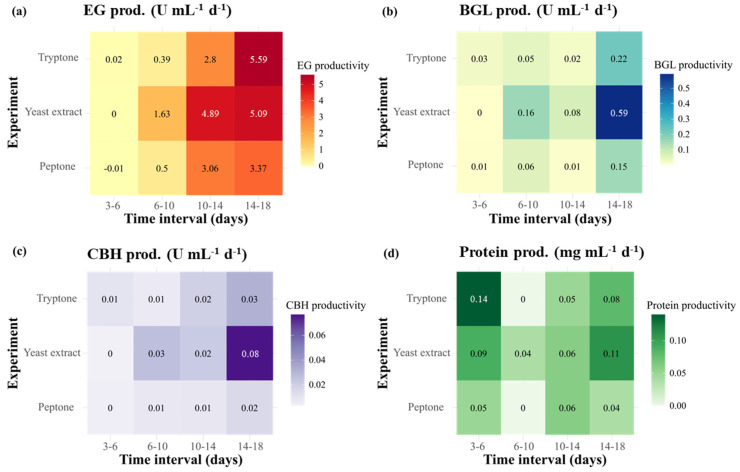
Time-interval productivity of enzymatic activities (EG, BGL, and CBH) and protein concentration of Mut-4 in response to different nitrogen sources. (**a**) EG (endoglucanase), (**b**) BGL (β-glucanase), (**c**) CBH (cellobiohydrolase), and (**d**) protein concentration.

The productivity in terms of enzymatic activities and protein concentrations over defined time intervals is presented in Figure 3. All experimental groups exhibited an initial lag phase up to days 10–14. However, the yeast extract group displayed faster and higher productivity compared with the remaining nitrogen sources during the early phase of cultivation. Over days 6–10, the yeast extract group showed higher EG productivity (1.63 U·mL^−1^·d^−1^) than did the tryptone and peptone groups (Figure 4a). Regarding BGL and CBH, the yeast extract not only yielded the highest productivity during this period (0.16 U·mL^−1^·d^−1^ for BGL; 0.03 U·mL^−1^·d^−1^ for CBH) but also reached peak levels over days 14–18, at 0.59 U·mL^−1^·d^−1^ for BGL and 0.08 U·mL^−1^·d^−1^ for CBH (Figure 4b,c). These values were approximately 2.7-fold higher for BGL and 2.7–4-fold higher for CBH than the values obtained for tryptone and peptone. Regarding protein production, the yeast extract also maintained a relatively stable productivity and reached a peak at 14–18 days (0.11 g·L^−1^·d^−1^), which was higher than that of tryptone (0.08 g·L^−1^·d^−1^) and peptone (0.04 g·L^−1^·d^−1^) (Figure 4d). Collectively, these results indicate that the yeast extract not only accelerated the early production of enzymes but also sustained a superior productivity for BGL and CBH at later stages compared with the remaining nitrogen sources.

This study demonstrated that the types of nitrogen source markedly impacted cellulase production by Mut-4. Yeast extract, tryptone, and peptone were selected as organic nitrogen sources because complex organics are known to support higher enzyme yields than inorganic salts [40,41]. These three components represent commonly used nitrogen sources with differing nutrient profiles. Yeast extract is a nutrient-rich preparation derived from autolyzed yeast cells containing abundant amino acids, vitamins, nucleotides, and trace minerals, whereas tryptone and peptone are protein hydrolysates, i.e., a casein enzymatic digest and a meat/gelatin digest, respectively, that mainly provide peptides and amino acids.

The use of this range of nitrogen sources allows the evaluation of the manner in which nutrient complexity affects fungal growth and enzyme synthesis. The yeast extract markedly enhanced early cellulase expression. During the early cultivation stage (6–10 days), yeast extract supplementation resulted in earlier and higher-level secretion of all cellulases, together with a pronounced increase in the production of all measured enzymes, compared with tryptone or peptone. These observations suggest that the yeast extract facilitated the transition out of the lag phase for enzyme synthesis. Its readily assimilable nutrients, including preformed amino acids and growth factors, likely enabled the rapid initiation of protein synthesis and enzyme secretion in the fungal cultures [41]. In contrast, tryptone and peptone, which lack the full spectrum of vitamins and trace nutrients, resulted in a slower start [42].

The yeast extract also led to the highest overall cellulase activity and protein yield. All maximum enzyme activities and the secreted protein concentrations were significantly greater in the yeast extract compared with the tryptone or peptone groups. These outcomes align with those of numerous studies showing that yeast extract is a superior nitrogen source for fungal enzyme production [41,42]. Maeda et al. (2010) demonstrated that the combination of urea and yeast extract significantly enhanced all cellulase activities in *Penicillium funiculosum*, achieving an increase of up to 6.7-fold in β-glucosidase activity [42]. Similarly, Gautam et al. (2010) reported that 1.0% yeast extract led to the highest production of all three major cellulase components in *Trichoderma viride* cultures, with activities increasing approximately by 4.0–5.9-fold compared with the lowest-performing conditions [41]. The enhanced performance of the yeast extract is likely attributable to its comprehensive nutrient composition and high bioavailability, which collectively promote fungal growth and efficient enzyme biosynthesis. Notably, the yeast extract exhibited a distinct advantage in promoting BGL production compared with tryptone and peptone. This may be attributed to the presence of specific vitamins or trace elements in the yeast extract that are necessary for BGL production [43]. As *Trichoderma* sp. typically produces BGL at relatively low levels, the ability of the yeast extract to overcome this limitation underscores the importance of nutrient complexity for achieving balanced cellulase induction [44].

Overall, these findings identify the yeast extract as the optimal nitrogen source for cellulase production in Mut-4, as it supported rapid initiation during the lag phase, afforded the highest enzyme yield, and sustained production into the late cultivation stage. These outcomes are consistent with previous reports highlighting the benefits of yeast extract for fungal enzyme production because of its comprehensive nutritional profile and possible inductive effects [26,45]. In contrast, the lower performances of tryptone and peptone further emphasize the importance of nutrient diversity. Media lacking the broad spectrum of growth factors present in the yeast extract are unlikely to support maximal enzyme production. Thus, the application of yeast extract represents a promising strategy for optimizing industrial cellulase production by fulfilling early-stage metabolic needs and sustained enzyme expression requirements.

### 2.3. Effect of Initial pH on Cellulase Activity

At an initial pH of 4.5, neither enzymatic activities nor protein concentrations were detectable throughout the cultivation period, indicating near-complete inhibition of cellulase secretion under acidic conditions. Therefore, pH 4.5 was excluded from comparative analyses. The time profiles of the enzymatic activities and protein concentrations under different initial pH conditions are presented in Figure S3. The profiles clearly showed a strong effect of the initial pH on cellulase production. In these profiles, pH 5.5 yielded a faster increase in enzyme activities and protein concentrations than pH 5.0. At pH 5.5, most enzymatic activities and protein concentrations rapidly increased and reached their maximum levels earlier (around day 14). In contrast, at pH 5.0, a delayed increase was observed, with peak values occurring later (around day 18). The maximum enzymatic activities and protein concentrations observed under the pH 5.5 and 5.0 conditions are summarized in Figure 1c and Table S1. At pH 5.5, the maximum activities of EG, BGL, and CBH reached 73.85, 4.21, and 1.11 U·mL^−1^, respectively, with a peak protein concentration of 1.41 mg·mL^−1^. The values observed at pH 5.5 were significantly higher than those obtained at pH 5.0 (*p* < 0.05), where all parameters decreased by approximately 16–38%. These trends suggest that maintaining an initial pH of 5.5 promotes not only a higher overall production but also an accelerated onset of cellulase secretion.

The maximum production rates of the enzymatic activities are presented in Figure 2c and Table S2. The values reported in Figure 2c were scaled using min–max normalization. At pH 5.5, the maximum rates were the highest, whereas those at pH 5.0 were lower, and no detectable rates were observed at pH 4.5. The maximum EG production rate observed at pH 5.5 was almost twice as high as that detected at pH 5.0. Similarly, the maximum BGL and CBH production rates at pH 5.5 (0.234 and 0.080 U·mL^−1^·d^−1^, respectively) were higher than those observed at pH 5.0, indicating that an initial pH of 5.5 was more favorable for both enzymatic activity and protein secretion.

**Figure 5:**
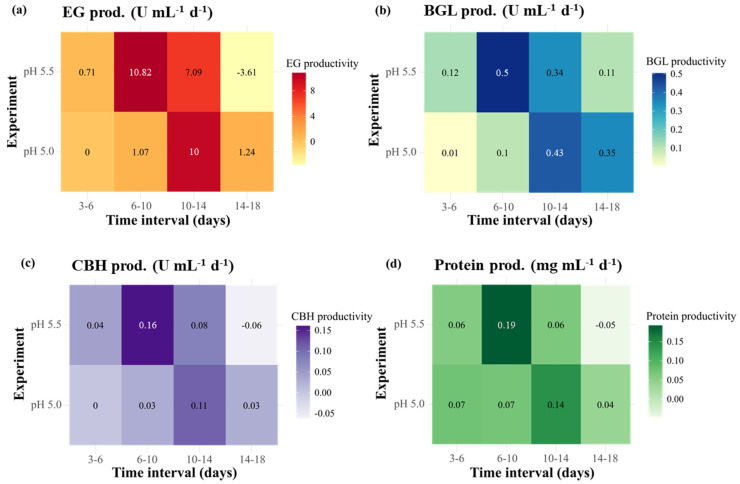
Time-interval productivity of enzymatic activities (EG, BGL, and CBH) and protein concentration of Mut-4 in response to different initial pH values. (**a**) EG (endoglucanase), (**b**) BGL (β-glucanase), (**c**) CBH (cellobiohydrolase), and (**d**) protein concentration.

The productivity regarding enzymatic activities and protein concentrations over different periods is presented in Figure 5. A higher early-phase productivity was observed at a pH of 5.5 compared with pH 5.0 across all parameters. At days 6–10, pH 5.5 exhibited significantly higher productivity than pH 5.0, with up to a 10-fold increase in EG, 5-fold in BGL and CBH, and nearly 3-fold in protein concentration.

These results suggest that an initial pH of 5.5 not only enhanced early cellulase and protein production but also contributed to the reduction of the overall culture period by accelerating enzyme secretion.

The effect of the initial pH on cellulase production in Mut-4 was evaluated using three representative pH values of 4.5, 5.0, and 5.5. This range was selected based on the well-established findings that filamentous fungi, including *Trichoderma* sp., typically produce cellulolytic enzymes most effectively under mildly acidic conditions, generally between pH 4.5 and 6.0 [38,41]. By selecting this pH range, this experiment aimed to determine the optimal conditions for maximal enzyme production while investigating how slight variations in acidity may affect (either positively or negatively) fungal physiology and metabolic activity.

At the lowest pH tested (4.5), cellulase activity and extracellular protein levels were entirely undetectable throughout the cultivation period. This outcome strongly suggests that the fungal cells experienced excessive acid stress, leading to the complete suppression of cellulase biosynthesis. Such conditions may compromise membrane integrity, denature secretory enzymes, and interfere with essential physiological processes, including nutrient transport, spore germination, and transcriptional activation [46,47]. Furthermore, exposure to acidic environments beyond the organism’s tolerance threshold may activate pH-responsive regulatory pathways, such as PacC/Rim101, leading to the downregulation of genes associated with extracellular enzyme production [48,49]. Taken together, these effects likely inhibited fungal growth or shifted the strain into a survival-oriented physiological state, thereby reducing or eliminating its metabolic capacity for enzyme secretion. In contrast, cultures initiated at pH 5.5 exhibited significantly enhanced cellulase production in magnitude and onset compared with those initiated at pH 5.0. Notably, enzyme secretion commenced earlier under pH 5.5, reaching its maximum yield by day 14, whereas cultures started at pH 5.0 exhibited a delayed peak production on day 18. These differences are likely attributable to the presence of a more favorable extracellular environment for fungal growth and enzyme activity at pH 5.5. Enhanced performance may result from reduced energy demands for acid stress adaptation and improved alignment with the optimal pH range of the secreted enzymes [50]. Furthermore, the ambient pH is a well-established modulator of gene expression; moderately higher pH conditions may relieve the repression of cellulolytic genes and facilitate transcriptional activation through pH-responsive regulatory pathways [49,51].

Beyond the differences in the final yields, the production rates obtained under the pH 5.5 condition were significantly enhanced. The rate of synthesis of EG, BGL, and CBH was notably faster at this pH value compared with pH 5.0, indicating that pH 5.5 not only increased the overall enzyme output but also accelerated the kinetics of enzyme biosynthesis. This improvement is likely attributable to a more rapid metabolic activation under the optimized pH conditions. In particular, the enhanced BGL activity observed at pH 5.5 may have played a critical role in mitigating the feedback inhibition caused by cellobiose accumulation, thereby promoting sustained enzyme expression and efficient substrate turnover [52].

The early-stage productivity observed under the pH 5.5 condition (days 6–10) further supports this trend. During this period, the production rates of EG, BGL, and CBH were markedly higher than those detected at pH 5.0. The earlier onset of enzyme secretion suggests a more rapid transition from vegetative fungal growth to the enzyme production phase. This shift may be attributed to improved hyphal development, enhanced substrate recognition, and more-efficient protein secretion mechanisms, all of which are favored under mildly acidic yet physiologically conducive conditions [53]. Moreover, the earlier activation of enzymatic activity may have accelerated the release of inducing sugars, thereby initiating a positive feedback loop that further amplified cellulase gene expression [54].

In summary, these experimental findings clearly demonstrated that an initial pH of 5.5 is optimal for cellulase production in Mut-4. This condition enabled earlier enzyme secretion, a higher maximum yield, increased production rates, and a superior early-stage productivity, which were in stark contrast with the complete inhibition observed at pH 4.5. The benefits of pH 5.5 can be attributed to an enhanced fungal metabolism, a greater enzyme stability, the relief of transcriptional and feedback inhibition, and an overall alignment between the environmental conditions and the functional requirements of the cellulase system.

### 2.4. Effect of Optimal Conditions on Cellulase Production in a Bioreactor

The bioreactor experiment was carried out to evaluate the effects of scaling up the optimal flask-level culture conditions. Based on the results of the previous optimization experiment involving the carbon source, nitrogen source, and initial pH value, the culture conditions including A3:C1 ratio as the carbon source and yeast extract as the nitrogen source were kept constant. The initial pH alone was adjusted to 5.5 for the optimized condition (OPT), whereas the original condition (ORI) was maintained at pH 5.0.

The time profiles depicted in Figure S4 illustrate the impact of the initial pH on enzyme production. The pH 5.5 culture experienced a shorter lag phase and a more rapid increase in cellulase activities compared with the ORI condition. Moreover, cultures performed under the ORI condition exhibited a prolonged adaptation period with gradual enzyme accumulation, whereas the OPT condition led to a quick shift into the exponential production phase, leading to a substantial enzymatic output within a shorter time frame. Notably, all enzymatic activities under the OPT condition exhibited a significant acceleration as early as day 6.

**Figure 6:**
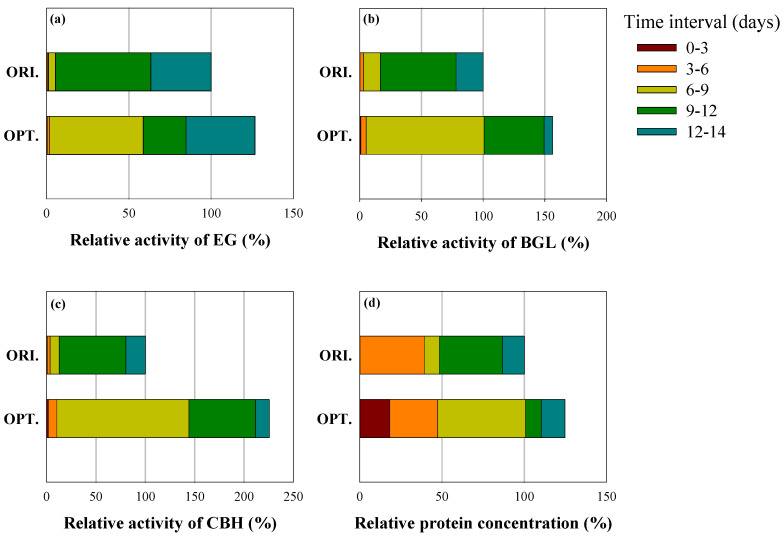
Relative activities in each time interval of cellulases (EG, BGL, and CBH) and protein concentration of Mut-4 under different values of initial pH at the reactor scale. (**a**) EG (endoglucanase), (**b**) BGL (β-glucosidase), (**c**) CBH (cellobiohydrolase), and (**d**) protein concentration. The initial pH is presented as follows: OPT. (pH 5.5) and ORI. (pH 5.0).

Figure 6 presents the relative activities (%) of EG, BGL, and CBH under each condition over time. Relative activity was calculated by dividing each activity value by the maximum value observed under the ORI condition and multiplying by 100; thus, the highest value in ORI was set as 100%. This representation enables a direct comparison of the activity progression under the two conditions. As shown in Figure 6, cultures that were initiated at pH 5.5 exhibited significantly higher cellulase activities than did those initiated at pH 5.0 throughout the culture period. Cellulase production also began earlier in the OPT culture, indicating a faster onset of secretion. Specifically, CBH activity was more than two-fold higher under OPT compared with ORI, whereas the BGL and EG activities were over 1.5-fold and 1.3-fold higher, respectively. These trends were particularly evident during the early-to-midlog phase (6–9 days), at which the relative activities observed under OPT consistently surpassed those detected under ORI. In addition to higher final activities, the early-stage productivity was markedly improved in the OPT culture. The sustained elevation in enzyme production observed under the optimized pH condition contributed to an increase in the overall enzyme yield and protein concentration. Collectively, these results suggest that adjusting the initial pH to 5.5 not only expedites the onset of cellulase production but also enhances the accumulation rate and overall productivity, thereby improving culture efficiency.

The cellulase yield achieved using Mut-4 under the optimized bioreactor conditions (EG, 46.54 U·mL^−1^; BGL, 3.71 U·mL^−1^; and CBH, 1.03 U·mL^−1^) greatly exceeded those reported in previous fungal cultivation. For example, Myeong et al. (2025) optimized a *Trichoderma* sp. KMF006 in a 10-L bioreactor and achieved maximum activities of only 33.60 U·mL^−1^ for EG, 3.46 U·mL^−1^ for BGL, and 0.63 U·mL^−1^ for CBH [22]. Similarly, de Carvalho et al. (2014) used *Penicillium funiculosum* ATCC11797 in a bioreactor with 10 g·L^−1^ Avicel as the carbon source and reported much lower activities (9.20 U·mL^−1^ for EG and 2.395 U·mL^−1^ for BGL; CBH was not detected) [55]. In all cases, Mut-4 delivered higher enzyme activities than those reported in previous reports. The EG activity of Mut-4 (46.54 U·mL^−1^) was approximately 1.39-fold that of KMF006 (33.60 U·mL^−1^) under comparable culture conditions. Similarly, the BGL and CBH activities of Mut-4 also exceeded those observed in KMF006. The study reported by Myeong et al. on *Trichoderma* sp. employed the same Avicel-to-cellulose ratio (A3:C1) used in the present study and found that this mixed carbon source accelerated cellulase production and reduced the overall cultivation time compared with cultures that used Avicel alone. By employing a carbon source consisting of 75% Avicel and 25% powdered cellulose (A3:C1), Mut-4 afforded a strong induction of cellulase synthesis while partially replacing the more expensive Avicel with a lower-cost cellulose alternative. In contrast, de Carvalho et al. used only Avicel as the carbon source. These results suggest not only that Mut-4 is inherently a high cellulase-producing strain but also that its combination with a mixed carbon source strategy affords the maximum enzyme productivity per unit of time.

Beyond the observed improvements in productivity, the overall cost efficiency of the process was also enhanced. The optimized cultivation of Mut-4 afforded high enzyme yields without the use of exotic supplements or excessively expensive inputs. Although the yeast extract is relatively expensive, it was applied at concentrations that were comparable to those used in previous studies, which also employed organic nitrogen sources. More importantly, the carbon source strategy adopted in this study helped reduce production costs by partially replacing the expensive Avicel with cellulose, which is a more economical alternative, thereby lowering the raw material cost per unit of enzyme produced. According to bioprocess modeling studies, the use of pure carbon sources, such as glucose or Avicel, may lead to the substrate accounting for >50% of the total cost of cellulase production [24]. Thus, the use of a mixed carbon source can significantly reduce the enzyme production costs by decreasing the reliance on expensive inducers. Ellilä et al. demonstrated that the use of low-cost residues, such as soybean hulls and molasses, as inducers of an engineered *Trichoderma reesei* can result in highly cost-effective cellulase production [56]. In agreement with this principle, the present study combined a high cellulase-producing strain with the partial substitution of expensive Avicel by low-cost cellulose, thereby enhancing the cost efficiency of the process.

In summary, the present study surpasses previous bioreactor-scale cultivation of fungal cellulase producers in terms of enzyme productivity and economic feasibility. The cellulolytic activities exhibited by Mut-4 surpassed those of previously reported strains under optimized submerged fermentation conditions. This enhanced volumetric productivity translates directly into a lower production cost per unit of enzymatic activity. Because enzymes often represent a major cost component in biomass conversions, improving enzyme productivity and reducing substrate costs are critical for overall process feasibility [57]. The use of a 3:1 Avicel-to-cellulose mixture in this study resulted in higher productivity compared with the pure Avicel feed cultivation reported in previous studies, while at least partially reducing the raw material costs. Thus, the Mut-4 process not only yielded a superior productivity but also exhibited a greater potential for cost-effectiveness compared with previously reported systems. These advantages highlight the feasibility of employing Mut-4 for industrial-scale cellulase production, thus contributing to reduced enzyme costs in lignocellulosic biorefinery applications.

## 3. Materials and Methods

### 3.1. Microorganism and Inoculum Preparation

A mutant strain of *Trichoderma* sp., designated Mut-4, was used in this study. This strain was generated previously through UV-induced mutagenesis by exposing a wild-type isolate to UV light at a distance of 4 cm for 4 min [31]. The ITS sequence of Mut-4 has been deposited in the NCBI GenBank database under accession number PV565723.

The Mut-4 strain was cultured on malt extract agar (MEA) plates containing malt extract (20 g·L^−1^), glucose (20 g·L^−1^), peptone (1 g·L^−1^), and agar (20 g·L^−1^) at 25 °C. Once the surface developed a dense green color, Mut-4 was subcultured and stored at 4 °C for preservation.

The preculture was prepared in a 250-mL baffled flask containing 100 mL of potato dextrose broth (PDB). The medium was sterilized by autoclaving at 121 °C for 20 min, then cooled to room temperature (25 °C). Fungal agar pellets (1 cm in diameter) of Mut-4 were cut from the MEA plates and 10 pellets were inoculated into the flask. The culture was incubated at 26 °C with shaking at 150 rpm for 5 days.

### 3.2. Effect of the Blending Ratio of the Carbon Source on Cellulase Production

Cellulase activity and productivity were evaluated in response to different blending ratios of the carbon sources using the OFAT method. The individual effect of the carbon source was investigated, while other parameters, such as the type of nitrogen source and initial pH, were fixed at yeast extract and pH 5.5, respectively.

Avicel and cellulose (Daejung Chemicals & Metals Co., Ltd., Siheung city, Republic of Korea) were used as the carbon sources at various ratios (Table 1). The crystallinity of each carbon source is presented in Table S3. All experimental groups were supplemented with a total carbon source concentration of 2% (*w*/*v*). The carbon source compositions were designated as A4:C0 (Avicel:cellulose = 4:0), A3:C1 (Avicel:cellulose = 3:1), and A0:C4 (Avicel:cellulose = 0:4), indicating the ratios of Avicel-to-cellulose supplemented in the medium.

The culture was carried out in 250-mL baffled flasks with a working volume of 100 mL. The basal medium consisted of yeast extract (10 g·L^−1^), KH_2_PO_4_ (5 g·L^−1^), K_2_HPO_4_ (5 g·L^−1^), MgSO_4_·7H_2_O (3 g·L^−1^), biochar (10 g·L^−1^), at an initial pH of 5.0. The medium was sterilized at 121 °C for 30 min. After cooling to room temperature (25 °C), 5% (*v*/*v*) of the inoculum was added. The flasks were incubated at 31 °C, which was selected based on our previous study, while shaking at 180 rpm for 18 days [23].

Samples were obtained by collecting 1 mL of the culture broth, which was subsequently centrifuged at 13,000 rpm for 10 min at 4 °C. The crude supernatant was then collected and stored at 4 °C for subsequent analysis. All experiments were performed in triplicate to ensure reproducibility.

### 3.3. Effect of the Type of Nitrogen Source on Cellulase Production

Using the OFAT method, the cellulase activity and productivity were assessed based on different nitrogen sources. The impact of each nitrogen source was examined, while the carbon source ratio (Avicel:cellulose = 3:1) and the initial pH value (pH 5.5) were kept constant.

Tryptone, yeast extract, and peptone were used as the nitrogen sources (Table 1). All experimental groups were supplemented with a total nitrogen source concentration of 1% (*w*/*v*). The carbon source composition was fixed at a 3:1 (*w*/*v*) ratio of Avicel-to-cellulose, with a total concentration of 2%. The initial pH of the medium was adjusted to 5.0. The medium composition, culture conditions, working volume, and sampling procedure were in accordance with the protocol outlined in Section 2.2. All experiments were performed in triplicate to ensure reproducibility.

### 3.4. Effect of Initial pH on Cellulase Production

The cellular activity and productivity were evaluated using the OFAT method with varying initial pH values. The effect of each initial pH value was investigated, while the carbon source ratio (Avicel:cellulose = 3:1) and nitrogen source (yeast extract) were fixed.

The initial pH value of the culture medium was adjusted to 4.5, 5.0, and 5.5 in the separate experimental groups (Table 1). The carbon source composition was fixed at a 3:1 (*w*/*v*) ratio of Avicel-to-cellulose, with a total concentration of 2%. Yeast extract was supplemented at 10 g·L^−1^ as the nitrogen source. The medium composition, culture conditions, working volume, and sampling procedure were in accordance with the protocol described in Section 2.2. All experiments were performed in triplicate to ensure reproducibility.

### 3.5. Evaluation of Cellulase Production in SmF Bioreactors Under Various Conditions

To evaluate the cellulase activity and productivity in the bioreactor system, cellulase production was carried out in a 10-L bioreactor with a working volume of 6 L. The medium consisted of Avicel (15 g·L^−1^), cellulose (5 g·L^−1^), yeast extract (10 g·L^−1^), KH_2_PO_4_ (5 g·L^−1^), K_2_HPO_4_ (5 g·L^−1^), MgSO_4_·7H_2_O (3 g·L^−1^), and biochar (10 g·L^−1^). Based on the results of previous experiments, the initial pH of the culture medium was adjusted to 5.0 and 5.5 in separate experimental groups (Table 1). The reactors were sterilized at 121 °C for 30 min and then cooled to room temperature (25 °C). Each reactor was inoculated with a 5% (*v*/*v*) preculture. The incubation conditions were set at 31 °C, with agitation at 180 rpm and aeration at 2 L·min^−1^ for 18 days. Sampling was performed according to the procedure described for the flask-scale experiments.

### 3.6. Assay Method

#### 3.6.1. Enzymatic Activity

The enzymatic activity of EG was evaluated using the Somogyi–Nelson method [58]. Carboxymethylcellulose (CMC, 2%) dissolved in 0.1 M sodium citrate buffer (pH 5.0) served as the substrate. A mixture of 45-μL CMC and 5-μL enzyme solution was incubated at 60 °C for 30 min. Subsequently, 50 μL of copper reagent was added, and the reaction was terminated by boiling for 10 min. The mixture was then treated with 50 μL of the Nelson reagent and diluted with 850 μL of distilled water. The absorbance of the supernatant was recorded at 650 nm.

For the BGL and CBH activity assays, 10 mM p-nitrophenyl-β-d-glycopyranoside (pNPG) and p-nitrophenyl-β-d-cellobioside (pNPC) were used as substrates, respectively, both of which were dissolved in 0.1 M sodium citrate buffer (pH 5.0). A reaction mixture comprising 160-μL buffer, 20-μL substrate, and 20-μL enzyme was incubated at 65 °C for 15 min. Then, 50-μL Na_2_CO_3_ was added, and the absorbance was measured at 405 nm [59]. One enzyme activity unit (U) was defined as the amount of enzyme required to release 1 μmol of glucose (EG) or p-nitrophenol (BGL, CBH) per minute. All measurements were performed in duplicate.

#### 3.6.2. Protein Concentration

The protein concentration was measured to assess fungal growth using a protein assay kit based on the Bradford method (Bio-Rad, Hercules, CA, USA). For the assay, a mixture containing 400 μL of distilled water, 100 μL of protein assay reagent, and 10 μL of enzyme solution was prepared and incubated at room temperature for 5 min. The absorbance of the mixture was recorded at 595 nm. The absorbance values were plotted against a standard curve that was prepared using bovine serum albumin. All measurements were performed in duplicate.

#### 3.6.3. Statistical Analysis

For each experimental condition, two independent cultures (biological replicates) were prepared. For each culture, the enzyme activity was measured in triplicate (technical replicates). Accordingly, the results were expressed as the mean ± SD (*n* = 6 per condition).

Statistical analyses were performed using the R software (v. 4.5.0). A multifactorial analysis was carried out to determine the significance of differences, with the significance threshold set at *p* < 0.05. Prior to applying the appropriate statistical methods, the normality and homogeneity of variance were tested. One-way ANOVA was performed to evaluate the differences among groups, followed by Tukey’s HSD test for post hoc comparisons.

### 3.7. X-Ray Diffraction (XRD) Analysis and Segal Crystallinity Calculation

X-ray diffraction (XRD) patterns were recorded in continuous scanning mode on an Ultima IV diffractometer (Rigaku, Tokyo, Japan) equipped with CuKα radiation (λ = 1.5418 Å, 40 kV, 40 mA) and a Kβ filter. The divergence slit was set to 1/2°, the divergence height-limiting slit to 5 mm, and both the scattering and receiving slits were kept open. Diffractograms were collected over a 2θ range of 5–40° with a step size of 0.02° at a scanning speed of 2°/min in a 2θ/θ configuration. Diffraction data were obtained from each carbon source samples (Avicel and cellulose) prepared under identical conditions. The crystallinity index (*CrI*) was calculated according to the Segal method by measuring the intensity of the (200) lattice plane (*I*_200_) and subtracting the amorphous contribution (*I_am_*) at approximately 2θ = 18°, after drawing a baseline between the lowest intensity points in the 2θ range of 7° and 37° [60].

(1) $$CrI=100\times \frac{\left(I_{200}-I_{am}\right)}{I_{200}}$$

## 4. Conclusions

This study demonstrated that cellulase productivity for lignocellulosic biomass conversion can be significantly enhanced by combining a high-performing mutant strain with systematically optimized parameters. The UV-induced mutant *Trichoderma* sp. Mut-4 was cultivated in a biochar-supplemented submerged fermentation system, in which the key parameters—carbon source composition, nitrogen source, and initial pH—were optimized to maximize enzyme productivity. The mixture of Avicel and cellulose (A3:C1) outperformed pure Avicel by promoting higher enzyme activity and accelerating secretion, thereby contributing to reduced raw material costs. Yeast extract was identified as the most effective nitrogen source, as it enhanced early-stage enzyme production and supported elevated overall yields. Moreover, an initial pH of 5.5 further improved the enzyme productivity and shortened the lag phase, underscoring the importance of maintaining a mildly acidic environment for optimal enzyme expression. Under these optimized conditions, Mut-4 maintained its high performance at the bioreactor scale, exhibiting greater EG, BGL, and CBH activities vs. previously reported strains under comparable conditions. These findings confirm the strong cellulolytic potential of Mut-4 and validate the effectiveness of the optimized SmF strategy for scale-up procedures.

This study identified the optimal culture conditions for Mut-4, a UV-induced mutant strain of Trichoderma sp. KMF006, within a newly developed biochar-supplemented SmF system, achieving high productivity without reliance on costly inducers. Collectively, these results represent a scalable and economically viable approach for industrial cellulase production, supporting the advancement of lignocellulosic biorefineries and contributing to the development of a sustainable bioeconomy.

## Figures and Tables

**Table 1:** Experimental conditions used for Mut-4 OFAT optimization.

Scale	Parameter	Experiment	Experimental Condition	
			Experimental Factor	Fixed Factor
Flask	Blending ratio of the C source	A4:C0	Avicel:Cellulose = 4:0	The N source is yeast extract and the initial pH is 5.0
		A3:C1	Avicel:Cellulose = 3:1	
		A0:C4	Avicel:Cellulose = 0:4	
	Type of N source	Tryptone	The N source is tryptone	The C source is A3:C1 and the initial pH is 5.0
		Yeast extract	The N source is the yeast extract	
		Peptone	The N source is peptone	
	Initial pH	pH 5.5	Initial pH is 5.5	The C source is A3:C1 and the N source is the yeast extract
		pH 5.0	Initial pH is 5.0	
		pH 4.5	Initial pH is 4.5	
Reactor	Initial pH	OPT.	Optimal condition (pH 5.5)	The C source is A3:C1 and the N source is the yeast extract
		ORI.	Original condition (pH 5.0)	

## Data Availability

The raw data supporting the conclusions of this article will be made available by the authors on request.

## Supplementary Materials

The following supporting information can be downloaded at: https://www.mdpi.com/article/10.3390/ijms26168000/s1.
