# Supplementary material for: Cellulase Production by Ultraviolet-Derived Mutant Trichoderma sp. Mut-4 Under Submerged Fermentation: Parameter Optimization and Large-Scale Application
Source: Int J Mol Sci. 2025 Aug 19;26(16):8000. doi: 10.3390/ijms26168000 (PMC12386674; doi:10.3390/ijms26168000)
Supplement: Supplementary file 1 [file ijms-26-08000-s001.zip › ijms-3736389-supplementary.pdf]

Supplementary Materials:

Table S1. Maximum enzymatic activities and protein concentration of Mut-4 in response to different optimization parameters.

Blending ratio of the C source[1)]	EG ($U\cdot mL^{-1}$)	BGL ($U\cdot mL^{-1}$)	CBH ($U\cdot mL^{-1}$)	Protein ($mg\cdot mL^{-1}$)
A4:C0	31.99 ± 2.89^{a}	2.78 ± 0.36^{a}	0.79 ± 0.24^{a}	0.98 ± 0.04^{a}
A3:C1	31.51 ± 4.67^{a}	2.97 ± 1.17^{a}	0.81 ± 0.05^{a}	0.87 ± 0.03^{ab}
A0:C4	28.03 ± 4.77^{a}	1.64 ± 0.69^{a}	0.44 ± 0.12^{b}	0.73 ± 0.11^{b}
**Type of N source**	**EG ($U\cdot mL^{-1}$)**	**BGL ($U\cdot mL^{-1}$)**	**CBH ($U\cdot mL^{-1}$)**	**Protein ($mg\cdot mL^{-1}$)**
Tryptone	35.27 ± 1.14^{b}	1.26 ± 0.23^{b}	0.26 ± 0.02^{b}	0.87 ± 0.05^{b}
Yeast extract	46.54 ± 2.71^{a}	3.33 ± 0.95^{a}	0.51 ± 0.14^{a}	1.09 ± 0.06^{a}
Peptone	27.84 ± 0.17^{c}	0.93 ± 0.04^{b}	0.14 ± 0.02^{b}	0.66 ± 0.03^{c}
**Initial pH**	**EG ($U\cdot mL^{-1}$)**	**BGL ($U\cdot mL^{-1}$)**	**CBH ($U\cdot mL^{-1}$)**	**Protein ($mg\cdot mL^{-1}$)**
pH 5.5	73.85 ± 5.63^{a}	4.21 ± 0.13^{a}	1.11 ± 0.11^{a}	1.41 ± 0.02^{a}
pH 5.0	49.34 ± 4.25^{b}	3.52 ± 0.36^{b}	0.69 ± 0.09^{b}	1.16 ± 0.03^{b}
pH 4.5[2)]	N/A	N/A	N/A	N/A

[1)] Blending ratio of the C source: the number represents the respective proportion of Avicel (A) and cellulose (C).

[2)] Owing to the lack of measurable activity at pH 4.5, data are not available and are indicated as N/A. Significant differences among the experimental conditions are indicated by different letters (P < 0.05).

Table S2. Maximum production rate of enzymatic activities of Mut-4 in response to different optimization parameters.

Blending ratio of the C source[1)]	EG ($U\cdot mL^{-1}\cdot d^{-1}$)	BGL ($U\cdot mL^{-1}\cdot d^{-1}$)	CBH ($U\cdot mL^{-1}\cdot d^{-1}$)	Protein ($mg\cdot mL^{-1}\cdot d^{-1}$)
A4:C0	1.777 ± 0.16	0.154 ± 0.02	0.056 ± 0.02	0.054 ± 0.00
A3:C1	1.750 ± 0.26	0.165 ± 0.06	0.058 ± 0.00	0.048 ± 0.00
A0:C4	1.557 ± 0.27	0.091 ± 0.04	0.032 ± 0.01	0.041 ± 0.01
**Type of N source**	**EG ($U\cdot mL^{-1}\cdot d^{-1}$)**	**BGL ($U\cdot mL^{-1}\cdot d^{-1}$)**	**CBH ($U\cdot mL^{-1}\cdot d^{-1}$)**	**Protein ($mg\cdot mL^{-1}\cdot d^{-1}$)**
Tryptone	1.959 ± 0.06	0.070 ± 0.01	0.015 ± 0.00	0.049 ± 0.00
Yeast extract	2.586 ± 0.15	0.185 ± 0.05	0.029 ± 0.01	0.060 ± 0.00
Peptone	1.547 ± 0.01	0.052 ± 0.00	0.008 ± 0.00	0.036 ± 0.00
**Initial pH**	**EG ($U\cdot mL^{-1}\cdot d^{-1}$)**	**BGL ($U\cdot mL^{-1}\cdot d^{-1}$)**	**CBH ($U\cdot mL^{-1}\cdot d^{-1}$)**	**Protein ($mg\cdot mL^{-1}\cdot d^{-1}$)**
pH 5.5	5.275 ± 0.40	0.234 ± 0.01	0.080 ± 0.01	0.101 ± 0.00
pH 5.0	2.741 ± 0.24	0.196 ± 0.02	0.038 ± 0.00	0.064 ± 0.00
pH 4.5[2)]	N/A	N/A	N/A	N/A

[1)] Blending ratio of the C source: the number represents the respective proportion of Avicel (A) and Cellulose (C).

[2)] Owing to the lack of measurable activity at pH 4.5, data are not available and are indicated as N/A.

Table S3. The crystallinity of Avicel and cellulose

	**Avicel**	**Cellulose**
**Crystallinity (%)**	75.521	53.995

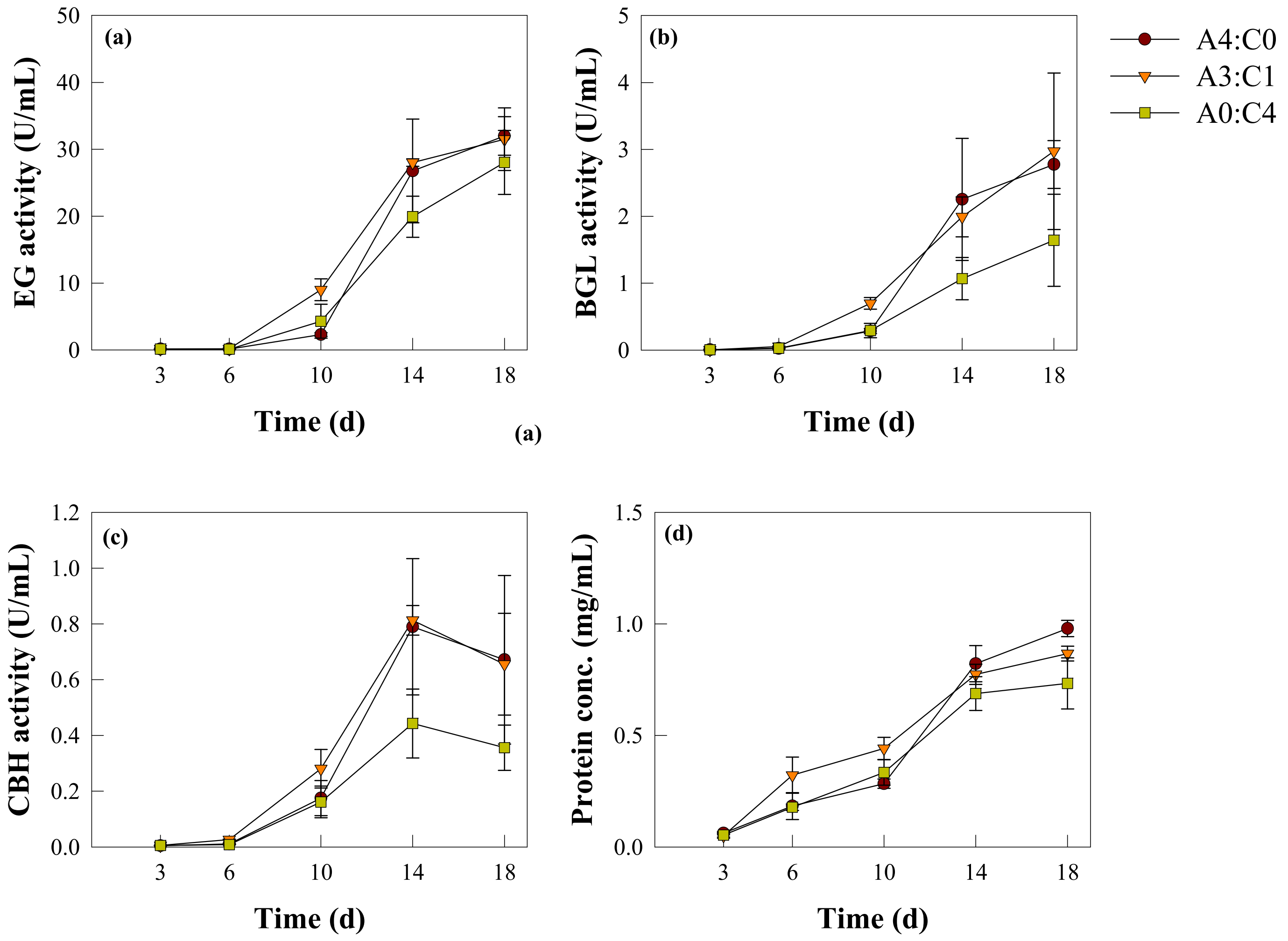

Figure S1. Time profiles of cellulase activities (EG, BGL, and CBH) and protein concentration of Mut-4 in response to different blending ratio of carbon sources. (a) EG (endoglucanase), (b) BGL (β-glucanase), (c) CBH (cellobiohydrolase), (d) Protein concentration. Blending ratios are repre-sented as follows: A4:C0, Avicel:Cellulose = 4:0; A3:C1 = Avicel:Cellulose = 3:1; A0:C4 = Avi-cel:Cellulose = 0:4.

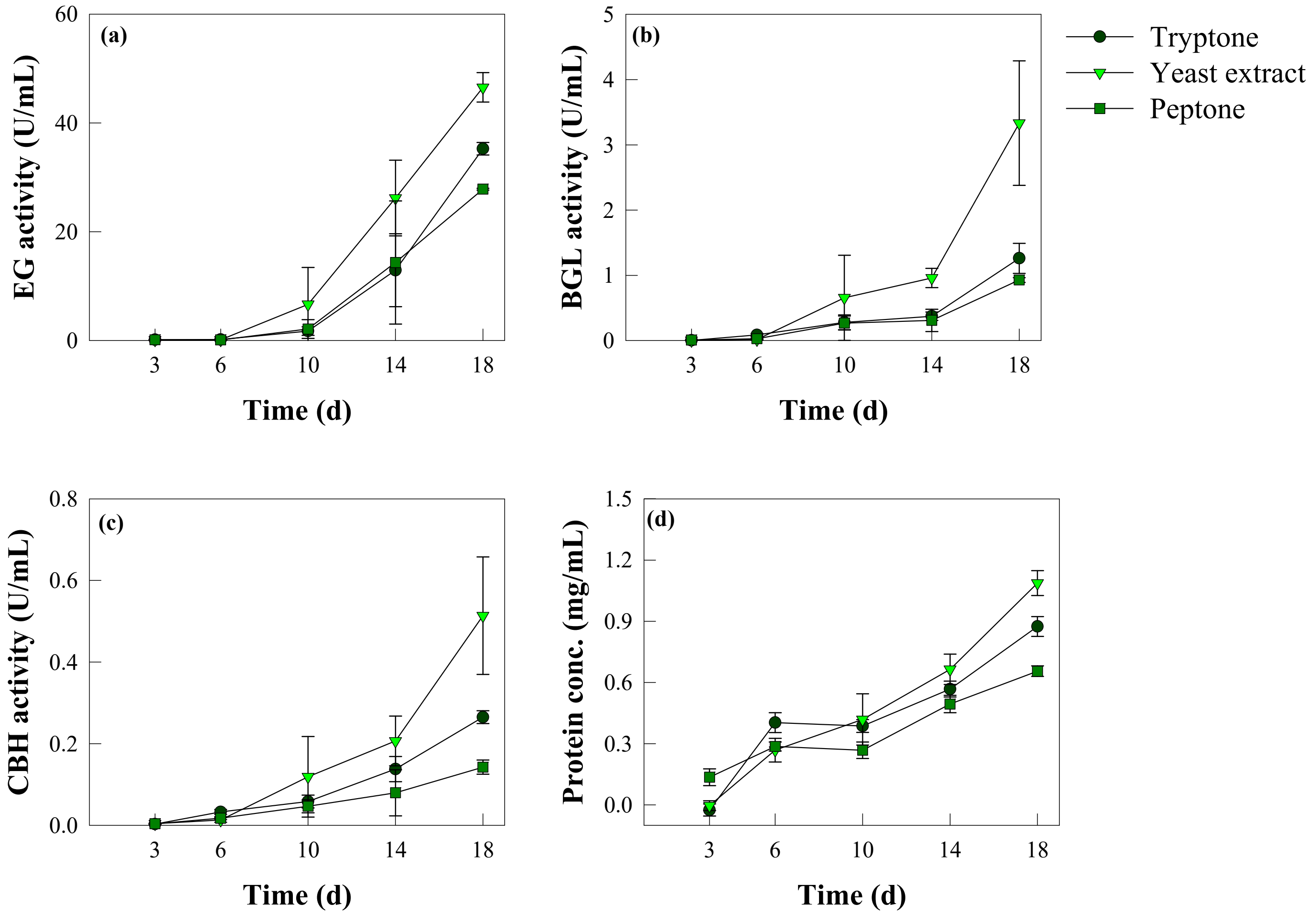

Figure S2. Time profiles of cellulase activities (EG, BGL, and CBH) and protein concentration of Mut-4 in response to different nitrogen sources. (a) EG (endoglucanase), (b) BGL (β-glucanase), (c) CBH (cellobiohydrolase), (d) Protein concentration.

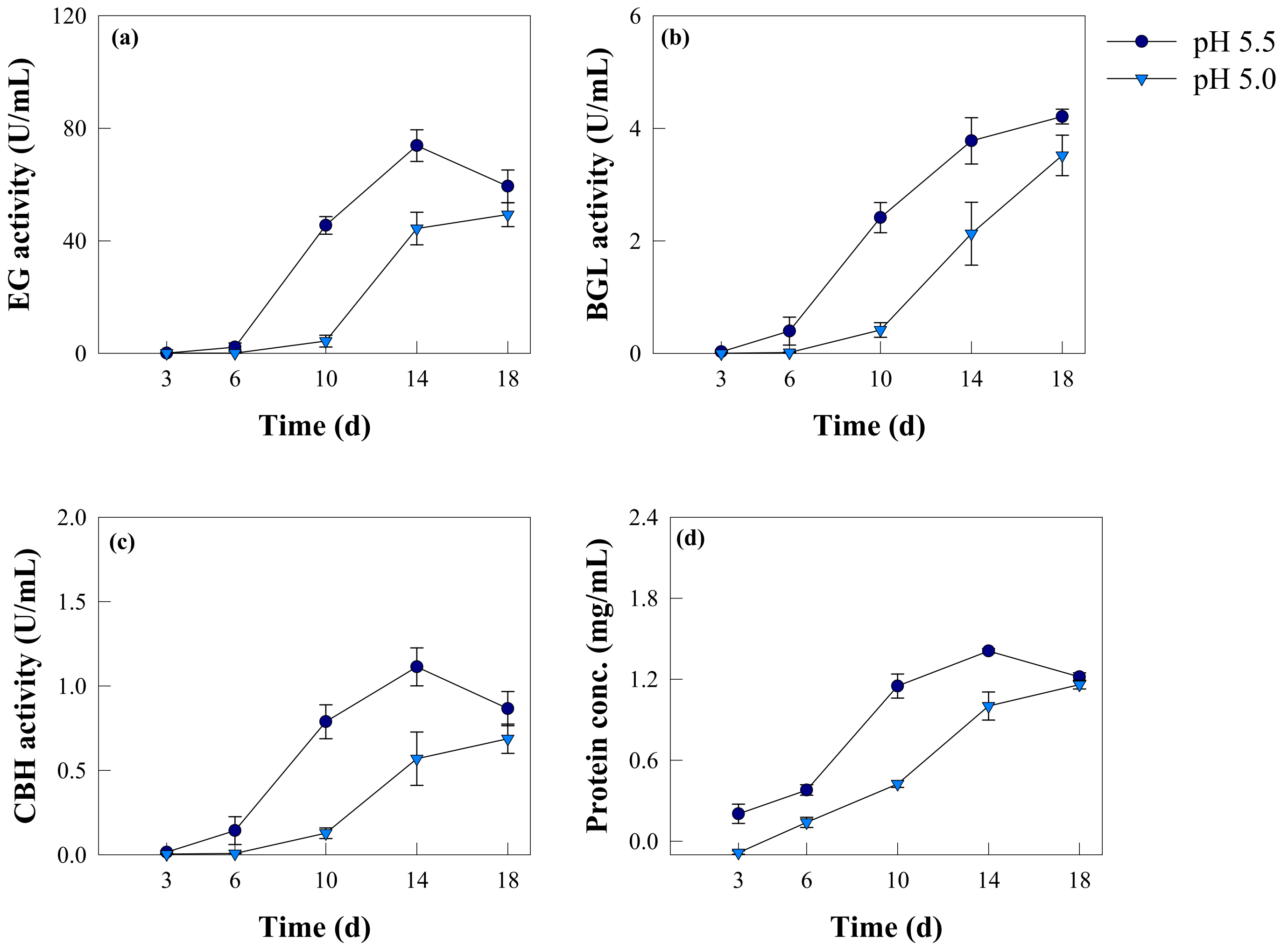

Figure S3. Time profiles of cellulase activities (EG, BGL, and CBH) and protein concentration of Mut-4 in response to different initial pH. (a) EG (endoglucanase), (b) BGL (β-glucanase), (c) CBH (cellobiohydrolase), (d) Protein concentration.

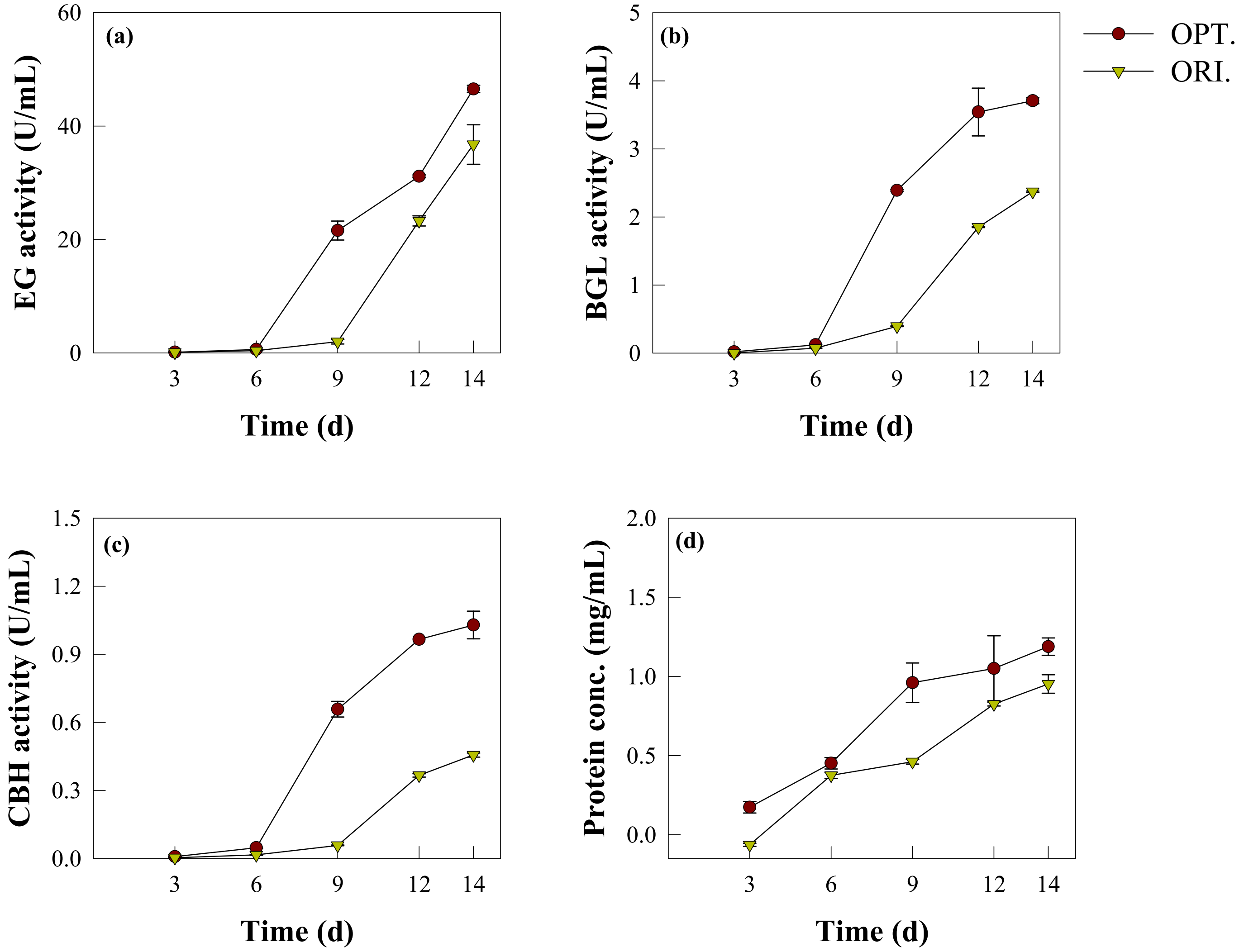

Figure S4. Time profiles of cellulase activities (EG, BGL, and CBH) and protein concentration of Mut-4 under different Initial pH in reactor scale. (a) EG (endoglucanase), (b) BGL (β-glucanase), (c) CBH (cellobiohydrolase), (d) Protein concentration. Initial pH is represented as follows: OPT. (pH 5.5) and ORI. (pH 5.0).
